# An Outbreak of Home Distillation Methanol Poisoning in Turkey During the COVID-19 Pandemic: A Single Center Experience

**DOI:** 10.34172/aim.2024.23

**Published:** 2024-03-01

**Authors:** Sertaç Güler, Dilber Üçöz Kocaşaban

**Affiliations:** ^1^Department of Emergency Medicine, University of Health Sciences, Ankara Training and Research Hospital, Ankara, Turkey

**Keywords:** Alcoholic intoxication, Distillation, Emergency medicine, Methanol, Outbreak

## Abstract

**Background:**

Causes of methanol poisoning may include accidental or suicidal use, as well as self home-distillation. In this study, it was aimed to evaluate clinical characteristics, laboratory findings, and outcomes of home-distillation methanol poisoning in two different time periods as an outbreak during the pandemic. The source of the methanol poisoning in all patients was home-brewing or distillation of methanol.

**Methods:**

The study was a single-center, retrospective, and observational case-control study. Patients over 18 years of age, in 2 different periods in the form of an outbreak due to home-distillation of methanol between April 1, 2020, and April 1, 2022, when the coronavirus disease 19 pandemic was intensely experienced in Turkey, were included in this study.

**Results:**

A total of 43 patients were included in the study. Of these patients, 22 were exposed to methanol between July and August 2020, and 21 patients were exposed to methanol in December 2021. Overall, 20 patients (46.5%) died, while 23 (53.5%) out of 43 patients recovered with or without sequelae. Patients with low blood pressure, oxygen saturation values, Glasgow Coma Scale (GCS) scores, high blood glucose levels, metabolic acidosis (pH<7.11), and high lactate levels (lactate>4.50 mmol/L) at admission had a statistically significantly worse prognosis.

**Conclusion:**

In methanol poisoning due to home brewing, low blood pressure, oxygen saturation, and impaired consciousness at the time of admission were clinical findings associated with mortality. In terms of laboratory findings, especially severe metabolic acidosis and lactate elevation were observed in the group that was mortal despite treatment.

## Introduction

 Methanol, also known as methyl alcohol, is a volatile and colorless substance. It is also known as wood alcohol since it is distilled from wood.^1,2^ Methanol is considered highly toxic and is generally only used for industrial purposes.^3^ Poisoning may occur orally, dermally, or by inhalation.^4^ The main metabolites responsible for toxicity in methanol poisoning are formaldehyde and formic acid, and patients usually experience symptoms such as nausea, vomiting, blurred vision, altered consciousness, dyspnea, and chest pain.^5^ Affected patients may experience mortality at a rate of up to 50%, and survivors may develop permanent visual and neurological impairments.^6,7^

 Methanol can be consumed as a cheap and illegal alternative to ethanol, especially in conditions where ethanol consumption is restricted or ethanol-containing alcoholic beverages with a banderol are heavily taxed or expensive.^3,5^ In this respect, deaths due to methanol poisoning are normally accidental, and it is rare to use methanol for suicidal purposes.^8^ This is mainly because it is a cheaper alternative to ethanol for alcohol production, and there is a tax obligation on products with banderol. For this reason, illegal or under-the-counter manufacturers put methanol on the market in packages that look like original alcohol bottles, and poisonings generally occur as a result of consuming these products.^5^ While a small number of sporadic cases can be found, methanol poisoning may occur in the form of epidemics from time to time in many countries around the world.^3,9^ The main causes of high mortality and morbidity in methanol poisoning, despite advances in treatment options, include late admission of affected people to healthcare for fear of being condemned or punished in places where alcohol consumption is unacceptable due to social or religious reasons, non-specific signs of poisoning, and the initial clinical situation resembling that of ethanol poisoning.^9,10^ The fact that laboratory tests such as the serum methanol level, format level, or osmolality analyses that may be required for a definitive diagnosis are not always available may make the diagnosis difficult.^11^

 During the COVID-19 pandemic, there was a period in which people were confined to their homes, social mobility decreased, curfews were present, and people consumed more alcohol due to the anxiety and fear of illness in Turkey and the rest of the world.^12^ For example, there have been massive methanol poisonings that may be associated with the pandemic in countries such as Iran.^2^ This may be related to the fact that methanol is cheaper than ethanol, and therefore fraudsters may have put methanol on the black market for home-made alcohol production and methanol contamination of some herbal distillation products.^2^ While methanol poisonings in the literature are generally due to accidental drinking, inhalation, or dermal exposure, the main difference and importance of our study is that poisoning in all patients was due to home-distillation methanol, and it was attempted to reveal its relationship with pandemic lockdown measures.

 In Turkey, cases of poisoning due to home-distillation of methanol have been increasingly observed in recent years, both due to the effect of the pandemic and for many other reasons, including the expensiveness of the drinks with the banderol or increased taxes. The other reasons are establishing alcohol distillation or brewing systems at home for hobby purposes, organizing parties or entertainment at home and accompanying them with alcoholic beverages, and the like.^13^

 This study sought to evaluate clinical characteristics, laboratory findings, treatment options, and outcomes of home-distillation methanol poisoning patients presented to our emergency department (ED) during two different time periods as an outbreak during the pandemic.

## Materials and Methods

###  Design and Patients

 Patients over the age of 18 who were diagnosed with methanol poisoning after admission to the ED of a tertiary training and research hospital due to drinking home-distillation alcoholic drinks between April 1, 2020, and April 1, 2022, when the COVID-19 pandemic was intense in Turkey, were included in the study. This was planned as a single-center retrospective observational case-control study. In Ankara, the capital city of Turkey, there were two methanol poisoning outbreaks due to drinking home-distillation drinks in July-August 2020 and December 2021. The hospital is a tertiary hospital with 800 beds and is one of the EDs in Ankara that accepts the most critically ill patients. It was found that alcohol was generally expensive in the country during these outbreaks, and the second outbreak was detected at a time when the new year was coming. Twenty-two patients were admitted to the ED between July and August 2020, and 21 patients were admitted in December 2021. Some patients were from the same family or friends who shared the same house.

###  Procedures

 Methanol levels cannot be measured in our hospital or other hospitals in our city. For this reason, the diagnosis of methanol poisoning is mainly based on a highly suspicious history (drinking home-distillation alcohol) and blood gas analysis (existence of increased anion gap metabolic acidosis and exclusion of other causes). The other diagnosis parameters were symptoms (alternation of consciousness, blurred vision, vomiting, headache, and chest pain) and negative blood ethanol levels. In addition, the fact that some of the patients were from the same family or the same house was important in terms of suspicious history, especially during such outbreaks.

 In addition to the demographic characteristics of the patients, vital signs, complaints, admission times, blood gas, and other laboratory results, the Acute Physiology and Chronic Health Evaluation (APACHE) II score and the Quick Sequential Organ Failure Assessment (qSOFA) score, which are predictive of mortality, treatment methods, the need for mechanical ventilation, and patient outcomes, were recorded on standard study forms. All examinations and follow-ups of the patients after their ED application were performed by emergency medicine physicians. Patients in need of mechanical ventilation and critical care services were followed up in the 10-bed emergency critical intensive care unit managed by emergency medicine specialists. Arterial blood gas, hemogram, cardiac markers (Creatinine kinase-myocardial band [CK-MB] and high-sensitive troponin), biochemical tests, coagulation parameters, and blood ethanol levels of all patients underwent investigation. Intracranial imaging was performed on all patients who had a change in consciousness or who developed a change in consciousness during the follow-up. Patients were divided into two groups according to their outcomes, including those who recovered with or without sequelae (Group 1) or exitus (Group 2). Mortality refers to mortality during the hospitalization process. The main outcome of the study was to determine the clinical and laboratory findings of the patients to predict mortality. Among the laboratory findings, especially the performance of blood gas parameters to predict mortality, was investigated.

###  Treatment

 Fomepizole or IV ethanol (10%) was administered to patients as an antidote treatment. Only 6 patients could be given fomepizole because of its high price, difficulty in obtaining it under pandemic conditions, and insufficient availability in the province. In patients in whom fomepizole could not be given, IV ethanol was administered to keep their blood ethanol levels between 100 and 150 mg/dL. A total of 30 patients were treated with 50 mg of IV folic acid (folate) every 6 hours. Hemodialysis was performed in all patients with high anion gap metabolic acidosis (pH < 7.30), blurred vision, or altered consciousness. Considering that the emergency critical intensive care unit was adequately equipped for hemodialysis, hemodialysis was applied in the intensive care unit, and then the patients were hospitalized and followed up there. Hemodialysis treatment was applied for at least two sessions of 4 hours or until the metabolic acidosis of the patients remained above 7.35 for at least 4 hours. Patients were taken to hemodialysis as quickly as possible after diagnosis. Sodium bicarbonate therapy was administered only to patients with severe metabolic acidosis (pH < 6.9 and HCO_3_ < 4 mEq/L) due to intracellular acidosis and free radical production. Extra-corporeal treatment could not be applied to our patients because there are no treatment facilities in our center.

###  Statistical Analysis

 The SPSS 22 package program (SPSS Inc.; Chicago, Illinois, United States) was used for the statistical evaluation of the data obtained from the study. The variables were expressed in two main groups, namely, categorical and continuous variables. Continuous variables are shown as means, minimums and maximums, *interquartile ranges*, and standard deviations. Categorical variables were expressed as numbers and percentages. The conformity of continuous variables to a normal distribution was calculated using the Kolmogorov-Smirnov and Shapiro-Wilk tests. Data that did not indicate normal distribution were compared through the Mann-Whitney U test and Kruskal-Wallis test, whereas data that demonstrated normal distribution were compared with the Student’s t-test. Pearson’s Chi-square and Fischer’s exact tests were utilized to compare the patients’ outcomes. The receiver operating characteristic (ROC) curves were used to compare blood gas parameters to predict mortality. The statistical significance level was considered to be *P* < 0.05.

## Results

 A total of 43 patients were included in the study. Of all patients, 22 were exposed to methanol between July and August 2020, and 21 of them were exposed to methanol in December 2021. When the patients were evaluated according to the outcomes, 23 recovered with or without sequelae (a total of 5 patients with sequelae, including 3 patients with visual sequelae and 2 patients with parkinsonism symptoms and memory deficits; Group 1), and 20 patients died (Group 2). The demographic characteristics of the patients, their complaints, and time of admission to the ED are provided in Table 1. Of all, 86% (n = 37) of the patients were male, and 14% (n = 6) were female. The mean age of all patients was 48.79 ± 12.19 (Min.-Max. = 22‒72). There was no significant relationship between the outcome of the patients and age, gender, or complaint of admission to the ED. No statistically significant difference was observed between the outcome of the patients and the time of admission to the ED (Table 1).

**Table 1:** Characteristics, Complaints, and Admission Time of Patients

	**Group 1 (n=23)**	**Group 2 (n=20)**	***P*** ** Value**
Age (years)	46.8 ± 12.9	51.0 ± 11.2	0.273
Females/males (*n*)	5/18	1/19	0.192*
Complaints^a^			
Changes of consciousness	12 (52.1)	17 (85.0)	0.083**
Visual disturbances	15 (65.2)	9 (45.0)	0.183**
Nausea/vomiting	15 (65.2)	8 (40.0)	0.098**
Dyspnea	7 (30.4)	9 (45.0)	0.324**
Chest pain	2 (8.6)	2 (8.6)	0.393**
Time of admission (After ingestion)^b^			
< 6 hours	5 (21.7)	7 (35.0)	0.096**
6‒12 hours	6 (26.0)	4 (20.0)	0.280**
12‒24 hours	10 (43.4)	6 (30.0)	0.316**
> 24 hours	2 (8,6)	3 (15.0)	0.588**

*Note*. Group 1 = Survivors with or without sequelae; Group 2 = Died. *Fischer’s exact test; **Pearson’s Chi-square test. ^a,b^Data are presented as n (%).

Table 2 presents the vital signs, Glasgow Coma Scale (GCS) score, and fingertip blood glucose mean values at the time of admission to the ED, according to the outcome of the patients. A statistically significant difference was found between patients discharged from ED with or without sequelae and patients who died in terms of systolic blood pressure (SBP), diastolic blood pressure (DBP), mean arterial pressure (MAP), and room air oxygen saturation values (*P* < 0.05). The prognosis of the patients with a lower GCS score at the time of admission was statistically significantly worse (*P* < 0.001).

**Table 2:** Means of Vital Signs, GCS Score, Fingertip Blood Sugar, and Cerebral CT of Patients According to Two Outcome Groups

	**Group 1 (n=23)**	**Group 2 (n=20)**	***P*** ** Value**
SBP (mm Hg), ± SD	129.1 ± 29.1	105.3 ± 43.7	0.047*
DBP (mm Hg), ± SD	77.9 ± 18.8	62.1 ± 24.6	0.025*
MAP (mm Hg), ± SD	94.9 ± 21.0	76.5 ± 30.5	0.030*
Pulse rate (beats/minute), ± SD	104.3 ± 27.5	99.3 ± 23.2	0.527*
Body temperature (°C), Min., Max.	36.4 (36.1‒36.9)	36.6 (36.2‒37.1)	0.456**
Oxygen saturation, Min., Max.	97 (93‒99)	90 (87‒97)	0.026**
GCS score, Min., Max.	13 (10‒14)	5 (3‒8)	< 0.001**
Fingertip blood sugar (mg/dL), ± SD	149.1 ± 46.3	192.3 ± 83.5	0.049*
Cerebral CT, total	23	20	0.528*
Cerebral CT, abnormal	1	8	0.007ᵠ

*Note*. Group 1 = Survivors with or without sequelae; Group 2 = Died. SBP: Systolic blood pressure; DBP: Diastolic blood pressure; MAP: Mean arterial pressure; GCS: Glasgow coma score; CT: Computed tomography; *Independent sample *t* test; **Mann-Whitney U test; ᵠFischer’s exact test; SD: Standard deviation; Min.: Minimum; Max.: Maximum.

 In addition, patients whose fingertip blood glucose was measured higher at the time of admission to the ED had a worse prognosis in terms of outcome (*P* < 0.05). All patients underwent cerebral computed tomography (CT). Cerebral CT results showed abnormal findings in a total of 9 patients (8 in the exitus group). Three patients had intracranial hemorrhage, and 6 patients had acute ischemic changes confirmed by diffusion magnetic resonance imaging results. Brain CT results included a statistically significant higher number of pathologies in the exitus group (*P* < 0.05).

 The laboratory values, the APACHE-II, and qSOFA scores of the patients at the time of admission are listed in Table 3. The APACHE II and qSOFA scores, which are among the mortality predictive scores, were statistically significantly higher in the deceased group (*P* < 0.001 and *P* = 0.005, respectively). The prognosis of the patients was statistically significantly worsened with an increase in lactate (*P* = 0.013), creatinine (*P* = 0.009), liver function tests (Aspartate aminotransferase, *P* = 0.002; Alanine aminotransferase, *P* = 0.002), amylase (*P* = 0.049), and troponin (*P* = 0.008). There was also a statistically significant difference between the groups in terms of the pH value (*P* = 0.016). The blood pH value was more acidic in group 2, which included patients who died, compared to group 1. There was no statistically significant difference between the groups in terms of bicarbonate, blood urea nitrogen, CK-MB, anion gap, calcium levels, hemogram parameters, and neutrophil-lymphocyte ratio (NLR).

**Table 3:** Laboratory Parameters and Mortality Predictive Scores of Patients at the Time of Admission

	**Group 1 (n=23)**	**Group 2 (n=20)**	***P*** ** Value**
pH, min-max	7.13 (7.01‒7.27)	6.92 (6.71‒7.14)	0.016*
HCO_3_ (mmol/L), Min., Max.	8.0 (5.2‒13.4)	5.95 (4.5‒10.7)	0.176*
Lactate (mmol/L), Min., Max.	3.9 (2.6‒7.0)	8.1 (4.9‒10.4)	0.013*
Anion gap (mEq/L), ± SD	5.40 ± 5.19	7.97 ± 3.85	0.297**
BUN (mg/dL), min., Max.	32 (20‒41)	30 (16‒70)	0.922*
Creatinine (mg/dL), Min., Max.	1.01 (0.81‒1.50)	1.36 (1.09-2.10)	0.009*
Amylase (U/L), Min., Max.	84 (65‒116)	124 (79‒184)	0.049*
AST (U/L), Min., Max.	27 (21‒41)	72 (31‒154)	0.002*
ALT (U/L), Min., Max.	14 (12‒26)	29 (23‒106)	0.002*
Calcium (mg/dL), ± SD	8.69 ± 0.77	8.49 ± 0.74	0.412**
hS-Trop T (ng/dL), Min., Max.	6.17 (4.3‒15.7)	15.9 (10.2‒105)	0.008*
CK-MB (µg/L), Min., Max.	4.21 (1.6‒7.1)	9.8 (3.7‒11.1)	0.100*
NLR, min-max	2.6 (2.0‒4.3)	4.7 (2.2‒8.6)	0.088*
Osmolarity (mOsmol/L), Min., Max	319 (302‒347)	321 (307-363)	0.559*
APACHE II, ± SD	20.04 ± 7.22	31.25 ± 57	< 0.001**
qSOFA, Min., Max.	1 (1‒2)	2.5 (2‒3)	0.005*

*Note*. Group 1 = Survivors with or without sequelae; Group 2 = Died. BUN: Blood urea nitrogen; AST: Aspartate aminotransferase; ALT: Alanine aminotransferase; HS-Trop T: High-sensitivity troponin T; CK-MB: Creatinine kinase-myocardial band; NLR: Neutrophil-to-lymphocyte ratio; APACHE: Acute physiology and chronic health evaluation score; qSOFA: Quick sequential organ failure assessment score; *Mann-Whitney U test; **Independent sample *t* test; SD: Standard deviation; Min.: Minimum; Max.: Maximum.

**Figure 1:**
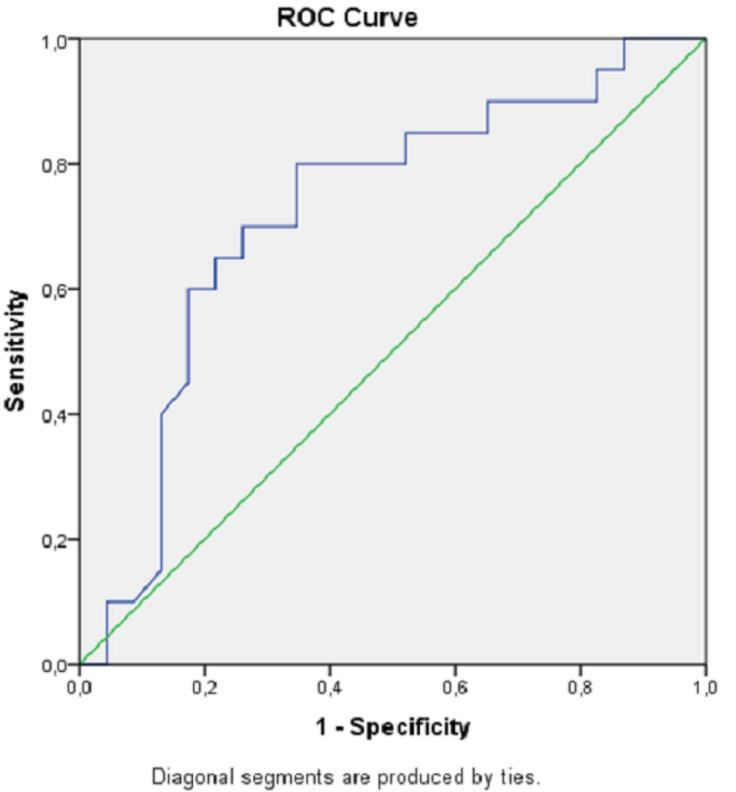
ROC Curve Showing Lactate in Predicting Hospital Mortality. *Note*. ROC curve: Receiver operating characteristic curve

**Figure 2:**
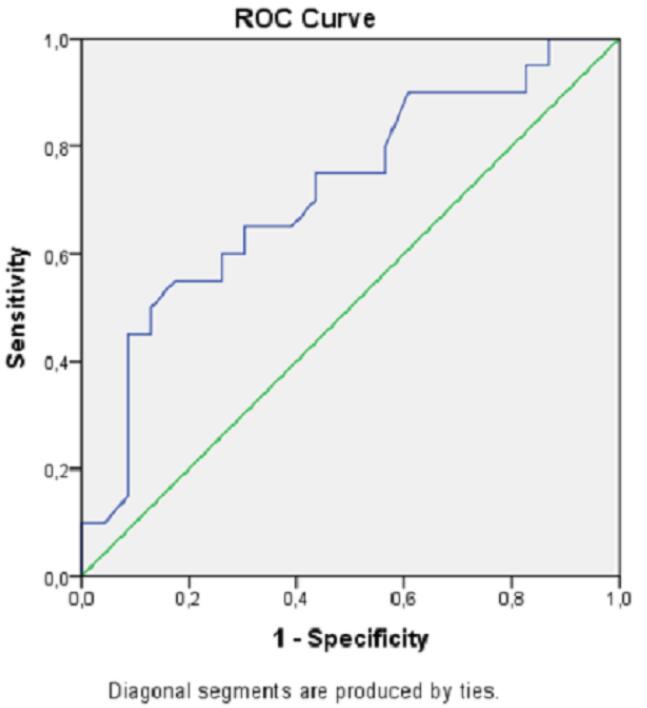
ROC Curve Displaying Blood pH in Predicting Hospital Mortality. *Note*. ROC curve: Receiver operating characteristic curve

 High lactate and low blood pH levels were associated with increased mortality. ROC curves for mortality estimation of the lactate level and blood pH value are shown in Figures 1 and 2, and analytical examinations are summarized in Table 4. Both parameters were found to be statistically significant in predicting mortality (*P*< 0.05). When the area under the curve (AUC) was examined, it was found that lactate level had a higher predictive power in the prediction of mortality (AUC = 0.72; 95% CI = 0.56‒0.88; *P*< 0.05). When the cut-off value was taken as 7.11 for pH, sensitivity and specificity were calculated as 75% and 56.5%, respectively. If the cut-off value was taken as 4.5 for lactate, sensitivity and specificity were estimated at 80% and 65.2%, respectively (Table 4).

**Table 4:** ROC Analysis of Lactate and Blood pH Levels for Hospital Mortality

	**AUC**	**CI 95%**	**Cut-off**	**Sensitivity**	**Specificity**	***P*** ** Value**
Lactate	0.722	0.563‒0.880	4.50	80.0	65.2	< 0.05
pH	0.714	0.558‒0.871	7.11	75.0	56.5	< 0.05

*Note*. AUC: Area under the curve; CI: Confidence interval.

Table 5 provides the treatments received by the patients during their stay in the ED and critical intensive care unit. There was no statistically significant difference between the death and survived groups in terms of ethanol/fomepizole, folate treatment, or hemodialysis treatments. In the group of patients who died, 2 cases could not be taken to hemodialysis because they were unstable when they were admitted to the ED and died until hemodialysis preparations were made. A statistically significant difference was observed between the groups in terms of mechanical ventilation needs. All the patients in the exitus group were connected to an invasive mechanical ventilator (*P*< 0.001).

**Table 5:** Treatment of Patients According to Outcome Groups

**Treatment Options**^a^	**Group 1 (n=23)**	**Group 2 (n=20)**	***P*** ** value**
Hemodialysis	23 (100)	18 (90.0)	0.420*
Ethanol	19 (82.6)	18 (90.0)	0.669*
Folate	16 (69.5)	14 (70.0)	0.975**
Mechanical ventilation	5 (21.7)	20 (100)	**<**0.001**
Fomepizole	4 (17.3)	2 (10.0)	0.458*

*Note*. Group 1 = Survivors with or without sequelae; Group 2 = Died. *Fischer’s exact test; **Pearson’s Chi-square test. ^a^Data are presented as n (%).

## Discussion

 Despite aggressive treatment measures, methanol poisoning is an important situation for emergency medicine physicians due to its high mortality rate.^1,3,9^ While methanol poisoning may be detected in fewer sporadic cases, it may also occur in the form of outbreaks in many countries around the world due to the illegal and mass production and sale of alcoholic beverages.^1,9,14,15^ The most important cause of methanol poisoning outbreaks is the fact that methanol is cheaper than ethanol. In this way, fake drinks produced illegally and put on the market with packages similar to the original packaging cause epidemics when consumed.^1,3,16^ High taxes or increased prices of original or labelled products may also direct people to cheaper options.^5,6,17^ Some of the methanol outbreaks are reported, but a significant portion is not reported; mortality rates exceed 30% in reported outbreaks, and significant morbidity is reported in survivors.^9^ In this study, we presented patients who were admitted to the ED after methanol poisoning due to home-distillation methanol during the pandemic in two different time periods as an outbreak. The main difference and characteristic of our study from the methanol poisoning outbreaks in the literature is that the cause of poisoning in all patients was home-distillation or brewing. To the best of our knowledge, this is the first study in the literature in which all patients were poisoned by the home-distillation of alcoholic drinks. Reasons for home-distillation alcohol may include cheaper alcohol consumption, the high cost of drinks with banderol and high taxes, and recently the establishment of alcohol distillation/brewing systems at home for hobby purposes.^13^ In addition, significant increases in methanol-related deaths and disabilities have been observed during the COVID-19 pandemic.^18-20^ One of the reasons for this is misinformation, especially from non-medical sources such as social media, that gargling or drinking alcoholic beverages would kill the virus.^19,21,22^ In addition, the increase in alcohol and substance use as a result of people spending more time at home and social isolation due to increased quarantine measures during the pandemic and efforts to control anxiety and depression with alcohol and/or other sedative drugs as a result of fear and anxiety of catching a disease or losing a relative due to illness may also play a role. Our study results were related to the adult population in Turkey who presented with methanol poisoning due to home-distillation methanol production during the pandemic. The same results cannot be generalized to poisonings associated with different countries, periods, ages, or methanol sources.

 In our study, patients with lower BP, oxygen saturation values, and GCS scores, higher spot blood glucose from the fingertip, metabolic acidosis (pH < 7.11), and high lactate value (lactate > 4.50 mmol/L) at ED admission were more likely to have a poor prognosis. Patients with higher liver function tests, creatinine values, and mortality predictive scores such as APACHE II and qSOFA had worse clinical outcomes.

 Our patients were generally middle-aged and mostly male, as in a similar study.^5^ The fact that alcohol abuse/addiction is generally a problem for men compared to women may cause such a result. Although the literature has shown that late hospital admission is associated with poor neurological and clinical outcomes in methanol poisoning,^5,16^ no statistically significant difference was found in our study in terms of the time of admission to the ED and the outcomes after methanol poisoning. This may be related to the number of patients in the study and the time intervals. The most common complaint of patients at admission to the ED was the altered level of consciousness and visual field defects, similar to the literature.^2,5,23^ This is generally due to the irreversible damage to the central nervous system and optic nerve caused by formic acid, which occurs as a result of the oxidation of methanol through the alcohol dehydrogenase enzyme.^1,5,10^

 Among the patients, those with lower BP and oxygen saturation in terms of vital signs had a statistically significantly worse prognosis. This may be explained by the irreversible effects of methanol on the respiratory and cardiovascular systems or by increased mortality in patients with respiratory distress. Again, the patients, who were found to have higher fingertip blood sugar at the time of admission to the ED, had a statistically significantly higher mortal prognosis. Adaptive stress hyperglycemia developed by the organism against fatal methanol poisoning may have caused this increased mortality.^5^ Patients with higher liver function tests, amylase, creatinine, and troponin values had a worse prognosis. This may again provide evidence that shock and related multi-organ failure have started in the group with higher mortality because the predictive scores of mortalities, such as qSOFA and APACHE II, which are frequently used in clinical practice, were also found to be higher on average in the same group. In addition, high liver and kidney function tests in the poor prognosis group may be explained by myoglobinuria, dehydration, or hypotension due to circulatory problems,^6,11^ and elevated cardiac markers may be explained by myocardial damage.^24^ Thus, arrhythmia and circulatory problems may have been observed more frequently in this patient group. In our patient group, we investigated whether NLR, one of the hemogram parameters that has a popular place in the emergency medicine literature, is of importance in terms of mortality. In our study, there was no significant difference between the groups in terms of NLR values. However, in a study evaluating 109 methanol poisoning patients, the researchers noted that NLR was able to distinguish death and survival with high performance, but it was ineffective in predicting vision loss.^25^ The researchers reported that the use of the platelet-to-lymphocyte ratio will provide better results than NLR in predicting permanent vision loss.^25^ There are also studies reporting that the red blood cell distribution width value, which is one of the hemogram parameters, is statistically significantly higher in patients who died from methanol poisoning compared to those who survived.^23^

 Brain CT results demonstrated immediate abnormal pathologies in 8 (40%) of our patients in the exitus group. This may be related to the increased sensitivity of the central nervous system to methanol.^5^ Histopathological examinations have shown cystic necrosis in the putamen, laminar necrosis in the cerebral cortex, and cystic necrosis in the cerebral white matter.^26^ These areas may be more susceptible to hypoxia and/or hypoglycemia or the accumulation of formic acid. It is thought that intracranial imaging should definitely be performed in patients who presented to the ED with the suspicion of methanol poisoning, especially if there is a change in consciousness. Moreover, since some of the patients admitted due to methanol poisoning may be chronic alcoholics, the change in their consciousness may not be obvious and may hide other symptoms. In our study, three patients had intracranial hemorrhage, and six patients had acute ischemic changes/diffusion limitations in different regions of the cerebral cortex.

 The main mechanism responsible for acidosis in methanol poisoning is the accumulation of formic acid in tissues. Afterward, formic acid suppresses the tissues’ use of oxygen and increases lactate production by causing anaerobic respiration. Formic acid and lactate together increase the anion gap.^5,16^ In our study, low pH values and increased lactate levels were found to be significant in demonstrating the severity of methanol poisoning, but there was no statistically significant difference between the two groups in terms of anion gap value. Additionally, the sensitivity and specificity of lactate and pH in predicting mortality were higher than the other parameters of blood gas in ROC analysis. As a result of decreased pH due to increased lactate production, the diffusion of formic acid through cell membranes becomes easier. This is the main mechanism responsible for central nervous system depression and hypotension in methanol poisoning.^5^ It is supposed that the fact that both SBP-DBP and MAPs were statistically significantly lower in the mortal poisoning group compared to the survivors in our study is related to this issue and, in a way, the amount of consumed methanol.

 Ethanol was generally used as an antidote treatment in patients rather than fomepizole due to its high price compared to ethanol and its limited availability, especially in pandemic conditions, and fomepizole could be given to a limited number of patients. In our study, no statistically significant relationship was found between the antidote or hemodialysis treatment and the outcomes of patients. Considering that the level of methanol could not be measured in our hospital, the duration of the dialysis sessions was decided mainly by looking at the improvement in metabolic acidosis. Hemodialysis sessions were terminated in patients whose blood gas pH was > 7.35 for at least 4 hours. The risk of shortening the dialysis duration of poisoned patients in the case of an outbreak was not mentioned in our study since the number of dialysis devices was enough.^5^ One of the factors that ensured this issue was that all the beds in the emergency critical intensive care unit, where we monitor patients, were suitable for hemodialysis facilities. Our patients were able to receive hemodialysis sessions lasting for about 4 hours, at least twice. Although it has been reported that folate treatment has a positive effect on mortality rather than visual sequelae after poisoning,^5^ such a relationship was not observed in our study.

## Limitations

 The main limitation of our study was the inability to measure the level of methanol in our hospital, province, and country. Therefore, the diagnosis was mainly based on a highly suspicious clinical history, symptoms, the exclusion of metabolic acidosis with an increased anion gap in blood gas and other causes, and ethanol negativity in blood biochemistry. In addition, it was not possible to use the required amount of fomepizole as an antidote treatment. The price and accessibility of the drug, as well as pandemic conditions, were the reasons for this limitation. If fomepizole could be used more liberally, a statistical difference could be detected between the groups in terms of treatment options. Apart from these, this was a single-center study, and extra-corporeal treatment options were unavailable in our center. This could have caused a potential bias because cases from other hospitals or regions might have different characteristics or outcomes, and different treatments across similar patients in other facilities could potentially influence outcomes. However, since hemodialysis is the main treatment option according to EXTRIP criteria,^27^ we do not think that the lack of other extra-corporeal treatment options such as hemofiltration or hemodiafiltration has changed the patient outcomes significantly. Furthermore, the anion gap of our patients was slightly higher than the normal value. We do not have a precise assessment of the cause of this situation. However, this may be related to the breakdown of methanol into formic acid, the toxic metabolite of methanol, and the time elapsed in this regard because about half of our patients presented to the ED more than 12 hours after poisoning.

## Conclusion

 Methanol poisoning is a poisoning method with a high mortality rate, and one of the reasons for this is the home-distillation of methanol. The reasons for home-distillation or brewing may include the fact that methanol is cheaper than ethanol, thus making alcohol cheaper, avoiding taxes and legal payments, trying to be different, or hobby purposes. Methanol poisoning that occurs in this way may occur as an outbreak when it concerns the same family and/or group of friends. In our study, patients with low BP, oxygen saturation values, and GCS scores, high spot blood glucose from the fingertip, metabolic acidosis (pH < 7.11), and high lactate values (lactate > 4.50 mmol/L) at admission to the ED had a worse prognosis. There was no statistically significant difference between the groups in terms of the complaints of the patients at admission to the ED, the time of admission, and treatment methods.
